# Supplementary figures and images for: Drosophila fabp is required for light-dependent Rhodopsin-1 clearance and photoreceptor survival
Source: PLoS Genet. 2021 Oct 29;17(10):e1009551. doi: 10.1371/journal.pgen.1009551 (PMC8580249; doi:10.1371/journal.pgen.1009551)

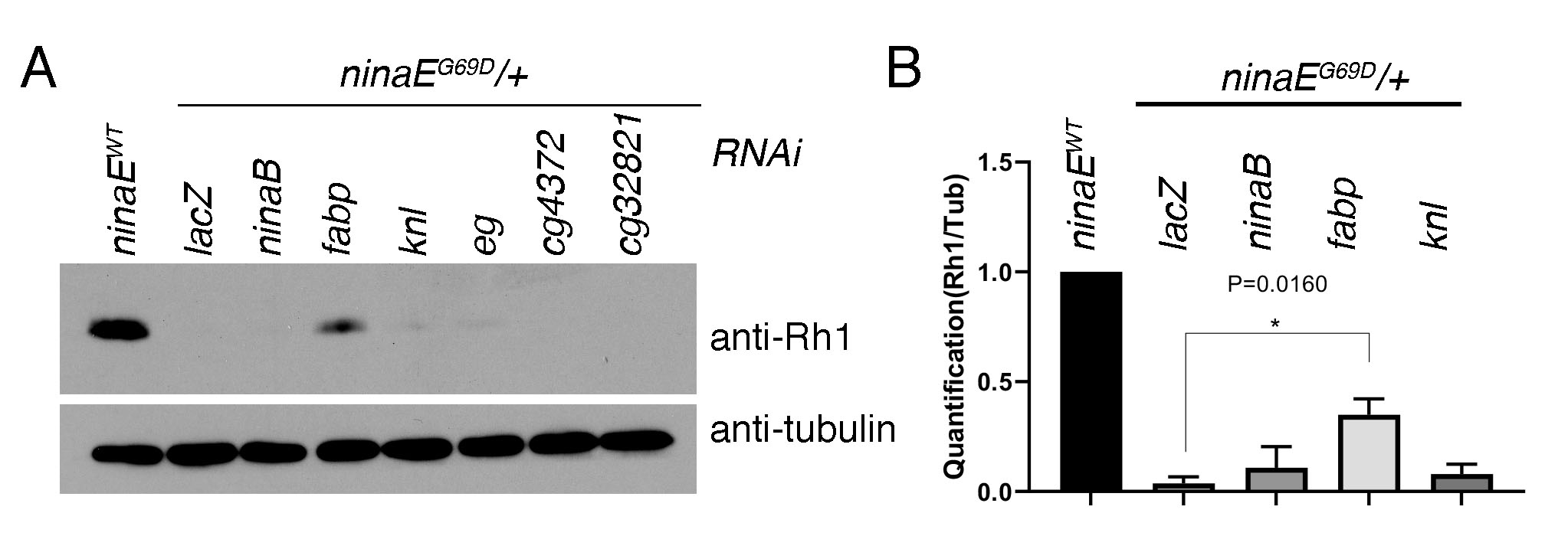

Supplement: S1 Fig — (A, B) Western blot of Rhodopsin-1 (Rh1) and β-tubulin from fly heads extracts. (A) The first lane is from ninaE wild type samples. The remaining lanes are from ninaEG69D/+ flies with the indicated genes knocked down through RNAi with the Rh1-Gal4 driver. lacZ RNAi (lane 2) was used as a negative control. (B) Quantification of relative Rh1 band intensities as normalized to β-tubulin. (JPG) [file pgen.1009551.s001.jpg]

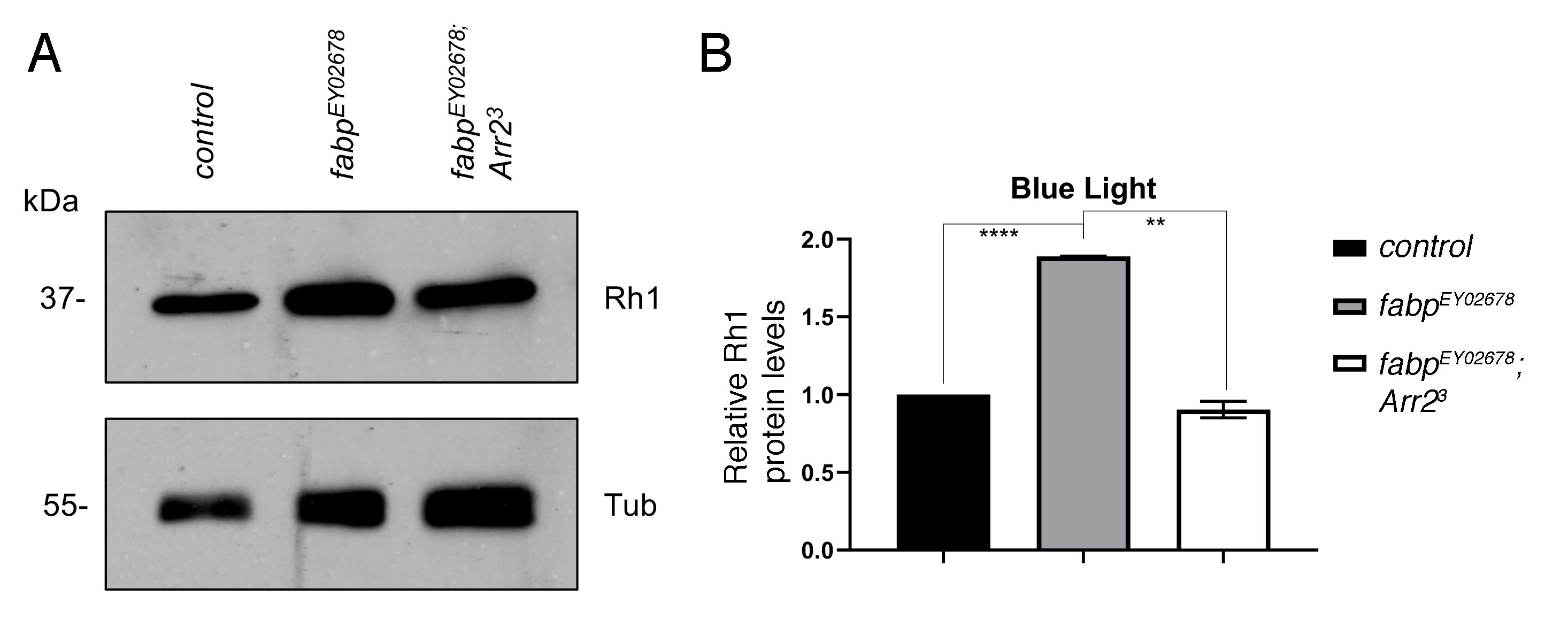

Supplement: S2 Fig — (A) Western blots of anti-Rh1 (top gel) and anti-β tubulin (bottom gel) in flies of the indicated genotypes that were exposed to blue light for 6 hours prior to analysis. (B) Quantification of Rh1 band intensities, normalized to that of β tubulin. The results indicate that fabp loss stabilizes Rh1 protein in flies exposed to blue light, and that function requires Arrestin2. Two tail t-tests were used to evaluate statistical significance. ** = p<0.005. **** = p<0.00005. (JPG) [file pgen.1009551.s002.jpg]

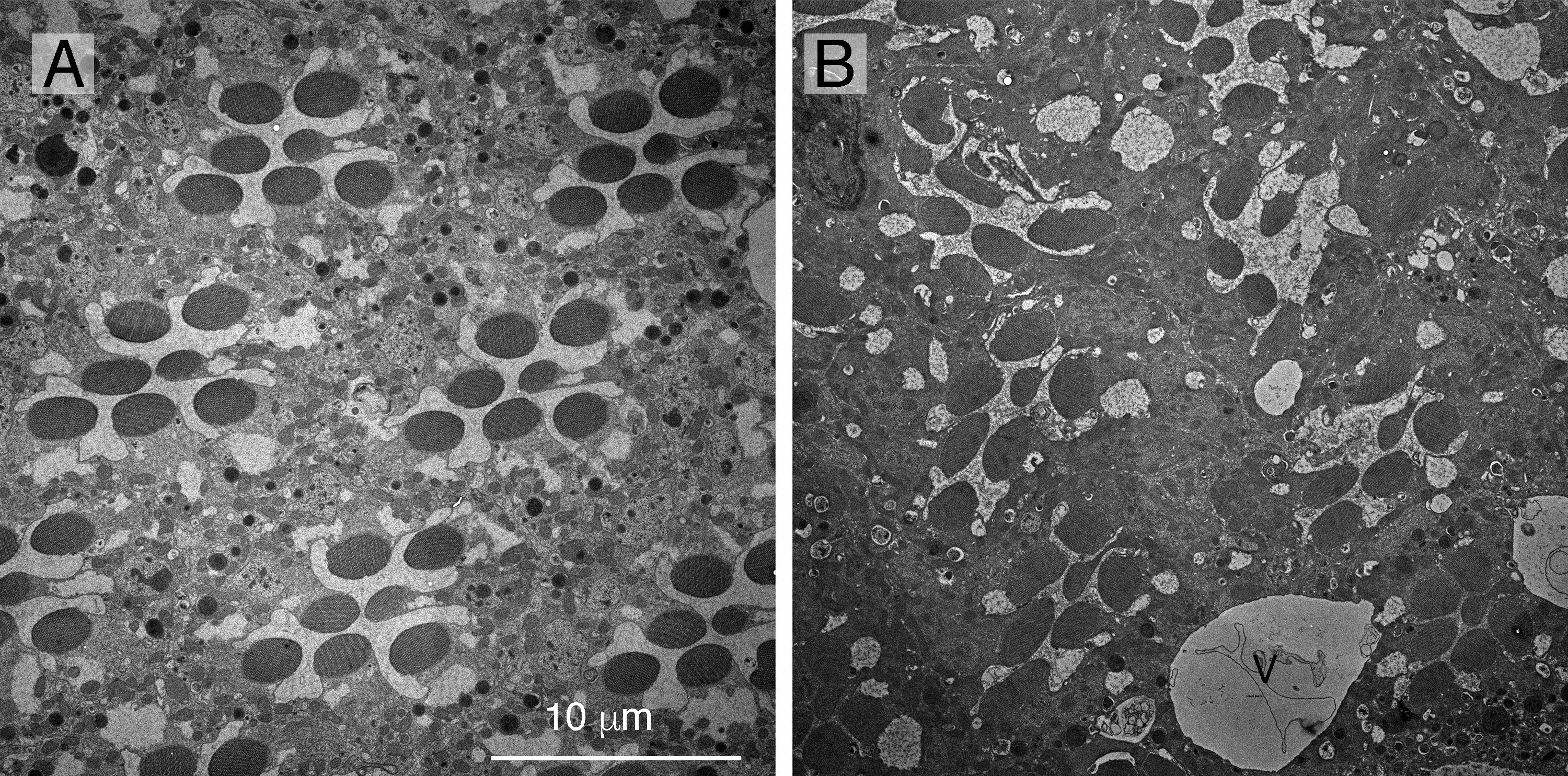

Supplement: S3 Fig — (A) Control fly eyes. Each ommatidium has seven rhabdomeres arranged in a trapezoidal pattern. These ommatidia are arranged in an array-like pattern throughout the adult eye. (B) Light-exposed fabpEY06747 -/- eyes at 27 days after eclosion. The ommatidial arrays show irregular patterns. Some ommatidia appear distorted, while others have missing rhabdomeres. There are vacuoles (marked with V) in between some ommatidia. (JPG) [file pgen.1009551.s003.jpg]

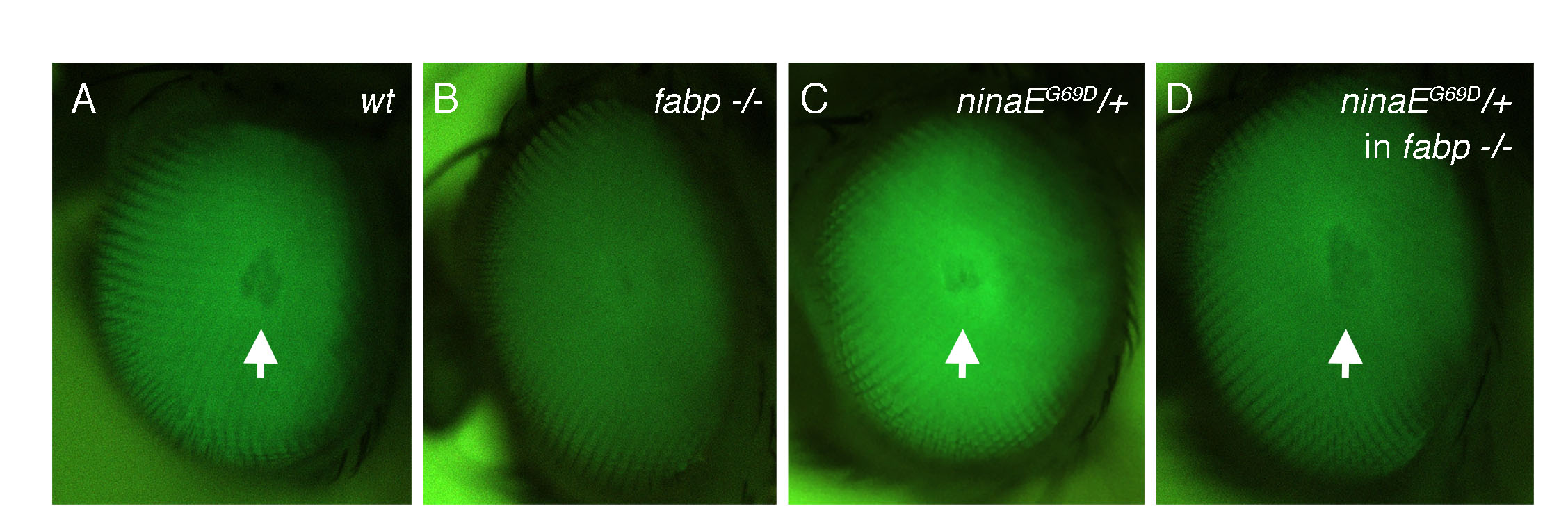

Supplement: S4 Fig — (A-D) Representative images of adult fly eyes of the indicated genotypes at 14 days after eclosion. White arrows point to the trapezoidal pattern of Rh1-GFP pseudopupils, which are indicative of intact photoreceptors. (JPG) [file pgen.1009551.s004.jpg]
