# Supplementary material for: Drosophila fabp is required for light-dependent Rhodopsin-1 clearance and photoreceptor survival
Source: PLoS Genet. 2021 Oct 29;17(10):e1009551. doi: 10.1371/journal.pgen.1009551 (PMC8580249; doi:10.1371/journal.pgen.1009551)
Supplement: Attachment — Submitted filename: fabp_rebuttal.pdf [file pgen.1009551.s007.pdf]

Point by point response

We thank the reviewers for carefully reviewing the manuscript. Overall, the reviewers commented that the study was interesting, but asked us to address questions regarding the mechanisms of *fabp* transcription regulation and regarding the Rh1 degradation pathway affected by *fabp*. We did our best to address these points in the following ways: (1) This revised manuscript now has significant amounts of new data regarding how *fabp* regulates Rh1 degradation. We now show that *fabp* is involved in a pathway that is initiated by Arrestin and regulated by Vps26. In addition, we improved the overall quality by adding new Transmission Electron Microscopy (TEM) images and double immuno-labeling results. (2) At the same time, the reviewer comments have made us realize that our original manuscript had too many speculative comments regarding retinoic acid-mediated gene expression control in *Drosophila*. Along these lines, we extensively revised the text to delete speculative statements, and to be more conservative in our data interpretation. Please note that we changed the title and the abstract of the manuscript, reflecting the changes in the manuscript. Below is a summary of the changes and our point-by-point response. Reviewer comments are in black font, and our responses are in blue.

Reviewer #1

Huang and Ryoo identified the fatty acid binding protein (fabp) gene was induced in ninaEG69D mutant fly eyes. Fabp is the fly ortholog of human CRABP-I, -II and FABP5. Among these human proteins, CRABP-I and -II have been shown to mediate retinoic acid signaling. Similar to CRABP-II, the authors showed that fabp expression could be induced by retinoid in cultured cells. They further demonstrated that a misfolding-prone rhodopsin-1 protein (ninaER69D) induced fabp expression, and loss of fabp alter photoreceptor functionality in vivo. This is an interesting study. Further mechanistic study, e.g. on the transcriptional regulation on fabp in the eyes and how fabp loss of function leads to photoreceptor malfunctioning should lead to a more thorough understanding of the role of fabp in Rhodopsin1 homeostasis.

Thank you for agreeing that our study is interesting. In response to the reviewer points, we have added new data that provides mechanistic details regarding how *fabp* loss increases Rh1 protein levels and promotes retinal degeneration (details are in the following section).

We feel that elucidating the precise mechanism by which retinoids regulate *fabp* transcription is beyond the scope of the current work. As you will see below (response to Reviewer #2), we have performed some retinoic acid binding studies to support our hypothesis, but to validate these ideas in vivo will require years of additional work. Therefore, we revised the text to limit any overstatements related to the role of retinoic acid signaling in gene expression control. Along these lines, you will see that we have revised the title, abstract and main text to highlight our focus on Rh1 protein homeostasis, and took out speculative comments about retinoic acid signaling.

Major comments:

Quality of data can further be improved

Please see that we have added a number of new image data in an effort to improve the overall quality of our manuscript (see below for details).

Why only 3 DAPI signal were detected in Fig. 1C. What were the other GFP-positive signals?

In the revised manuscript, we replaced the image in Fig. 1C with that with more DAPI nuclei. These nuclei are associated with the anti-GFP beads (green). We updated the Figure legend accordingly.

It is not clear how the authors could differentiate properly folded Rhodopsin-1 protein over misfolded Rhodopsin-1.

Our inference to misfolded Rh-1 is based on the induction of ER stress-inducible markers by the mutant Rhodopsin-1 gene. We acknowledge that this is only an indirect evidence for protein misfolding. To be more conservative with the use of terms, we now delete the term "misfolding-prone Rhodopsin-1" in the Abstract, and "misfolded Rhodopsin-1" in the page 5. We replaced these terms with description like, "encoded Rh1 protein imposes ER stress" (Abstract), or "these mutations impose stress in the ER" (in page 5).

Did the authors observe induction of fabp protein in S2 cells? Also, was Fabp protein induction observed when wildtype flies were treated with RA?

We now include anti-FABP western blot results from S2 cells in the revised Figure 2C, D, which shows a moderate increase in FABP levels after 3 hours of RA treatment.

We were not able to see FABP induction when we fed flies with RA, but this could be due to technical reasons (e.g. insufficient RA uptake in vivo, or only moderate FABP induction that couldn't be distinguished through stainings).

While we think FABP being inducible by RA is highly interesting, we also acknowledge that we do not have strong evidence that RA acts as a signaling molecule that actively regulates gene expression. Therefore, we took out all text that could be criticized as misleading. Among others, we now write in the Discussion that "there is as yet no evidence to support the existence of a retinoic acid signaling pathway in *Drosophila* similar to those delineated in vertebrates" (page 19). We also re-wrote the Abstract and the Introduction, taking out parts that highlight the possibility of RA signaling pathway conservation in *Drosophila*.

Had the ninaD experiment been performed (Figure 3) in fly eyes?

In Figure 2E - I, we showed the effect of ninaD loss in fly head gene expression.

Did the authors observe induction of fabp-GFP genetrap expression in the eyes of ninaEG69D mutant?

> In the revised Figure 1G and H, we now show that the endogenous FABP protein increases in *ninaE*^{G69D}/+ fly heads. We hope the reviewer agrees that showing endogenous FABP protein increases is equivalent to (or perhaps even better than) using a protein trap reporter line.

Was RNAi gene knockdown and overexpression effect confirmed by RT-PCR and/or Western blot (Figure 4)?

> For many transcripts, RT-PCR from fly head samples is not appropriate to assess in vivo knockdown efficiency. This is because we are specifically knocking down genes of interest in R1 to R6 photoreceptors (using Rh1-Gal4), while the genes of interest may be also expressed in other cell types of the fly head. Therefore, we state in the revised manuscript that "it is possible that the negative outcome is due to insufficient RNAi knockdown efficiency, or perhaps because those genes act outside the photoreceptor cells where the RNAi lines were expressed" (page 10, 11). Please note that we moved the RNAi data from Figure 4 to Supplementary Figure 1 because of the inconclusive nature of the results. We hope the reviewer agrees that the negative results from RNAi should not detract from the main focus of this manuscript, which is to present evidence that *fabp* affects light-dependent Rh1 clearance.

Error bars are missing in Figure 6F and G.

> We repeated the retinal degeneration experiments in the original Figure 6F, G. We now present the results in the form of a Kaplan-Meier curve, and Log-rank tests were used to assess statistical significance (see revised Figures 7E, F).

Minor comments:

Quality of some figures can further be improved.

> In response, we replaced the old Figures 1B, C, and added new Transmission Electron Microscopy (TEM) images in Figure 6.

Quantification of eg, cg4372 and cg32821 is missing in Figure 4B.

> As we stated above, we do not want to detract the readers with negative results from RNAi experiments. The reason for showing those RNAi results was to explain how we became interested in *fabp*. To help focus more on *fabp*, we moved the initial RNAi screening result to the Supplementary Figure 1 in the revised manuscript. The revised Figure 4 now focuses on the characterization of

fabp. If reviewer feels that the RNAi data detracts from the main message of the manuscript, we could consider taking out those results from the manuscript.

Labeling is missing in Fig. 4D.

Thank you for pointing this out. The labels have now been added.

Figure 5: Endosomal vesicle marker should be included to demonstrate the identity of the green signals in Fig. 5F.

In the revised Figures 6F and G, we provide double labeled images with endosomal markers Rab5 (early endosomes) and Rab7 (late endosomes). We write that there is a partial overlap (not complete), and conclude that Rh1 localize to several different intracellular sites.

Beyond the co-localization experiments, we also added genetic interaction results in the revised Figure 4, supporting the role of *fabp* in the regulation of light-dependent Rh1 endocytosis and degradation.

Molecular weight ladder and scale bar are missing in some blots/gels (Fig. 2A/C) and confocal micrographs (e.g. Fig. 1B and C) respectively.

We have now added a scale bar for Figure 1, and molecular weight labels for Figures 2, 4 and 5.

Spelling mistakes are found in some figures (e.g. Figure 4E).

Thank you for catching this. We fixed what was written before as “antRh1”.

It is not clear which fabp mutant was used in Figure 5.

We specified in the Figure 5 legend that *fabp EY02678* alelle was used.

Line172 ninaE wild type flies: when describing wildtype flies, “ninaE” can be omitted.

Following the suggestion, we deleted the word “ninaE” from that sentence.

Reviewer #2:

In this manuscript, Huang and Ryoo identified fabp increased in ninaEG69D by photoreceptor-specific gene expression profiling. The authors found that fabp is a retinoid-induced gene, and functions in Rh1 hemeostasis and photoreceptor cell integrity. Moreover, loss of fabp could suppress the retinal degeneration of ninaEG69D. The author claimed that retinoid-inducible gene expression program might regulates Rh1 proteostasis and photoreceptor survival. Overall it is an interesting and important work.

Thank you for agreeing that our work is interesting.

However, the manuscript lacks detail mechanisms. The authors showed that loss of fabp caused an accumulation of Rh1 proteins in cytoplasmic vesicles, but failed to give a convincing explanation.

We have added additional data in the revised manuscript to provide cell biological insights regarding the *fabp* mutant phenotype. Specifically, we show in the revised Figure 5C, D that increases in Rh1 levels caused by *fabp* loss is almost completely reversed in *Arrestin2; fabp* double mutant flies. We also added new data in Figure 5E, F that *Vps26* overexpression suppresses the *fabp* mutant phenotype. *Arrestin2* and *Vps26* are genes previously reported to regulate light-activated Rh1 levels in endosomes (Dolph et al., 1993; Alloway et al., 1999; Wang et al., 2014). Together with the fact that *fabp* mutant phenotypes manifest only when flies are reared under light (Figure 5A), these new data indicate that *fabp* is involved in Arrestin-mediated Rh1 degradation.

The authors showed that fabp is upregulated by ninaEG69D and/or retinoid acid, but did not provided by which pathway (for exmaple, ER stress) fabp is upregulated. It has been reported that mutations in fatty acid transport protein (fatp) caused adult-onset and progressive photoreceptor cell death through increasing Rh1 levels (PMID: 22844251), the phenotype is similar as fabp mutants, FABP is simple downstream of FATP or not.

We have now added genetic interaction results with *Arrestin2*, *Vps26* and *fatty acid transport protein*. The results indicate that *fabp* affects Rh1 clearance through the Arrestin2-mediated endosomal pathway (see below for more details).

Major points

It is important to check if FABP can bind to retinoid and which species retinoid it binds. If the retinoid levels and ratio is changed in fabp mutants. These biochemical characteristics might help to explain the genetic phenotypes of fabp mutants.

In response to this point, we performed in vitro Fabp – retinoic acid binding experiments (shown below):

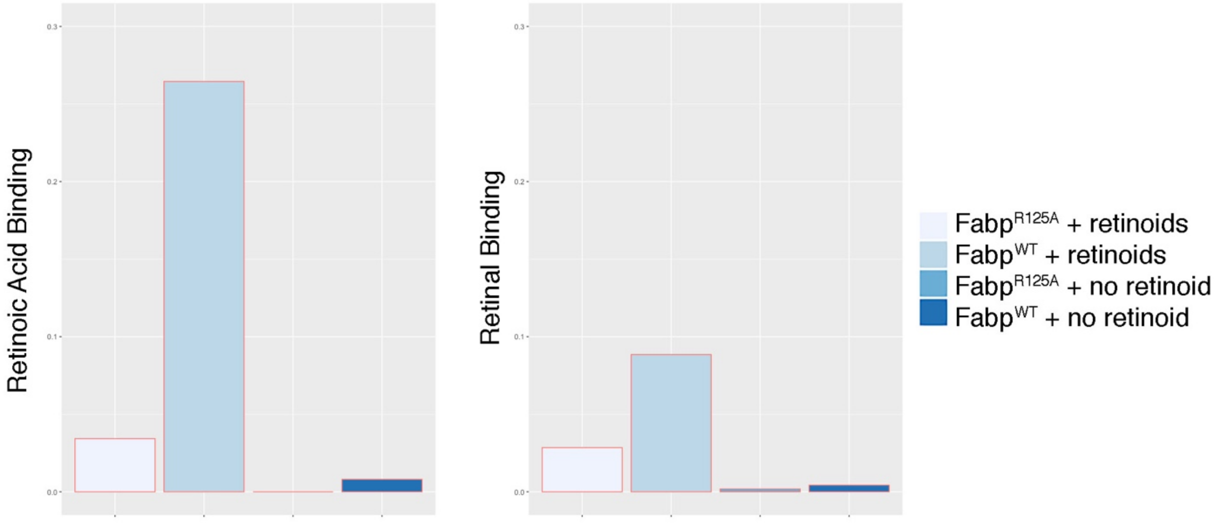

Specifically, we incubated recombinant GST-Fabp^{WT} and mutant GST-Fabp^{125A} with or without a retinoid cocktail (mixture of retinoic acid and retinal). Fabp^{125A} serves as a negative control, as it is equivalent to a mammalian CRABP mutant that fails to bind retinoic acids. We then ran the samples on native PAGE gels to purify the recombinant proteins. The gel fragments were analyzed through mass-spec to quantify the retinoid species that co-purified with the recombinant proteins. The results indicate that recombinant Fabp^{WT} retains retinoic acids with higher yield than Fabp^{125A}. Retinal was also retained by Fabp^{WT}, but to a lesser degree than Fabp^{WT}.

While these results are interesting to us, we feel that additional experiments are required to establish that Fabp binds to retinoic acids in vivo, and to determine the physiological significance. Because a thorough analysis of Fabp – retinoic acid interaction is really beyond the scope of this current work, we deleted all references in the manuscript regarding FABP's potential ability to bind to retinoids. As you will see, we revised the manuscript extensively to focus on the idea that *fabp* affects light-activated Rh1 degradation, thereby affecting retinal degeneration.

Authors first found that fabp transcription is induced by ninaEG69D, then they found that the it is inducible by Retinoic acid. The link between these two regulations is weak. The free retinoid levels might explain the phenotypes.

We fully acknowledge that we do not know how retinoids affect *fabp* expression. As we wrote in our comments to Reviewer 1, we took out text that may mislead the readers into thinking that *fabp* is induced by a specific RA signaling pathway analogous to that of mammals. Along these lines, we now write in the Discussion that "there is as yet no evidence to support the idea that retinoic acids regulate gene expression in *Drosophila* through specific pathways similar to those delineated in vertebrates" (page 19). We also re-wrote the Abstract and the

Introduction, taking out parts that highlight the possibility of RA signaling in *Drosophila*.

In mammals, retinoic acid serves as an important signaling molecule, but flies lack canonical retinoic acid signaling. The author found that fabp is regulated by retinoid acid, but there are not evidence supporting the transcriptional pathway that retinoid acid regulated in flies. Consider it is induced by misfolded Rh1, retinoic acid might induce ER stress response, which upregulates fabp.

We agree that we have no evidence of *Drosophila* having canonical retinoic acid signaling mechanisms, and made statements along those lines in the revised Discussion section (see above).

Mutations in fatty acid transport protein (fatp) caused adult-onset and progressive photoreceptor cell death through increasing Rh1 levels (PMID: 22844251). This retinal degeneration is also suppressed by the ninaEG69D. It is important to check if FABP and FATP acts in same pathway and the epistasis of fabp and fatp in rhodopsin homeostasis.

Following the reviewer suggestion, we performed experiments where we overexpressed *fabp* in the *fatp* knockdown background, or conversely, *fatp* overexpression in the *fabp* mutant background. However, these experiments did not support an epistatic relationship between the two genes (revised Figure 5E, F). We decided not to draw major conclusions based on these negative results, because there is no evidence that overexpressing *fabp* or *fatp* is sufficient to stimulate Rh1 degradation through the endosome (which is a pre-requisite for examining an epistatic relationship).

The reviewer may have asked about the relationship between *fatp* and *fabp* to understand which genetic pathway is associated with *fabp*-mediated Rh1 clearance. This question is addressed in our revised Figure 5D, where we show that Rh1 accumulation in *fabp* mutants requires *Arrestin2*. In Figure 5E, F, we also show that *Vps26* (another regulator of Rh1 at endosomes) genetically interacts with *fabp.* These data indicate that *fabp* affects the endosomal Rh1 degradation pathway.

Loss of increase fabp could increase rhodopsin levels in ninaEG69D/+ flies. It is assumed that the Rh1 that detected is wild-type form, which can explain the fact that degeneration can be rescued. It is important to have experimental evidence to support it.

This is a valid point. To address this, we added new data using a Rh1 transgene in which the wild type Rh1 is tagged with an HSV epitope. Using this line, we show in Figure 4F that the wild type Rh1 levels increase significantly in *fabp* mutants in the *ninaE G69D/+* background.

Deep pseudopupil assay can reflect multiple defects, including Rh1 homeostasis, compactness of rhobdomere structure and cell viability. The retinal degeneration needs to be examined by TEM or other proper assays to support the conclusion.

> Following the reviewer suggestion, we now add TEM images of *fabp* mutant eyes in Figure 7A-C and Figure S3.

The authors showed that fabp is retinoid-inducible, and found that it is requried for Rh1 homeostasis. however, if induction of fabp by retinoid is important for Rh1 is not convincing. Loss of fabp increased Rh1 levels in ninaEG69D and suppressed its retinal degeneration. As the retinoid regulated gene, it assumes that mutations disrupting retinoid metabolisms such as ninaD and santamaria downregulates fabp and have same effects as fabp mutations. However, in figure 4A, RNAi against ninaB seems did not affect the Rh1 in ninaEG69D.

> As the reviewer writes, we obtained negative results with *ninaB* RNAi. But we do not think we could draw positive conclusions based on such negative results. The most likely reason that Rh1 levels are not affected by *Rh1-Gal4* driven *ninaB* knockdown is that we knocked down this gene specifically in the photoreceptor cells, while it had been established that ninaB acts outside the retina (Pak, 1979; von Lintig and Vogt, 2000 PMID 10766819; Kiefer et al., 2001 PMID 11278918). Thus, we did not take account the *ninaB* negative results into formulating a conclusion. Instead, we relied on two independent positive results based on *ninaD* and *santa maria* mutants. To avoid detracting the readers from *ninaB* RNAi results, that data has been moved to the Supplemental Figure 1.

Minor points:

Authors used GFP trap lines to examine the response of FABP to retinoid in vivo. The major signals are detected in the gut, which is different with its endogenous function that authors proposed. The retina/photoreceptor cell signals should be checked.

> In the revised Figure 1G, H, we now show that FABP protein levels increase in *ninaE*^{G69D}/+ head extracts. In addition, Figure 2E, F show that FABP protein levels decrease in *ninaD* and *santa maria* mutant fly heads. We hope the reviewer agrees that following the changes of endogenous FABP protein is equivalent to, or perhaps even better than, analyzing an FABP protein trap reporter.

Fig. 4D missing labels.

> Thank you for catching our mistake. The label has been added.

The MW markers should be indicated in Figs 2A, 2C, 4A, 4D, 4E and 5A.

> Thank you for pointing this out. We’ve now added the molecular weight markers.

Fig. 5 D-G need a quantification. As the Rh1 levels increased, the pictures that only showed increased vesicle fluorescence is not convincing. A endosomal marker need to be used if authors claimed that these are endosomal vesicle.

To address this point, we provide additional western blot data in Figures 5C and E, and quantified the increase in Rh1 levels in the newly added Figures 5D and F. We hope the reviewer agrees that western blot results are more suitable for quantification than imaging results. To assess endosomal localization of Rh1, we now add new data with early and late endosomal markers, Rab5 and Rab7 (Figure 6F, G). There is a partial, but not a complete, co-localization. We suggest that Rh1 in distributed in multiple intraceullar sites (which is not surprising given the complexity of the endosomal trafficking pathway). At the same time, we added new genetic interaction results supporting the idea that *fabp* acts in a pathway that involves *Arrestin2* and *Vps26* (Figure 5C-F). We hope the reviewer agrees that these data collectively strengthen our main model.

Reviewer #3:

The study by Huang and Ryoo investigates retinoid-inducible genes in Drosophila, which have been poorly studied. They report a novel role for fabp a retinoid-inducible gene in Rh1 regulation and retinal degeneration. By performing a gene profiling, they first identified fabp, among others, as a gene which expression is increased in ninaEG69D/+ mutant compared to wild type retina. They next show that fabp levels are sensitive to retinoic acid treatments and mutant affecting retinoic acid metabolism. The loss of fabp function analysis revealed that Rh1 levels are upregulated in fabp mutant both in wild type and ninaEG69D mutants. Presumably fabp is required for lysosomal/endosomal Rh1 degradation in light dependent manner, shown by the accumulation of Rh1 vesicules that accumulate upon light in fabp mutants. They finally show that double mutant fabp and ninaEG69D have reduced degeneration compared to single mutants.

This is an original study which presents interesting and novel results. Most of the results are supported by data.

We thank the reviewer for the positive assessment.

I have some comments that may help to further improve the manuscript in particular about the mechanism regulating Rh1 upregulation in fabp mutant and the cause of retinal degeneration in fabp mutant. There is also a possible issue with the use of Rh1 driver in ninaEG69D mutant in Figure 1 and 6 that needs to be addressed.

-Regarding the method used for gene profiling I have an issue with the fact that Rh1-GAL4 was used to drive the expression of GFP:MSH in ninaEG69D, in which Rh1 transcription is upregulated. The authors need to comment on this and in particular if this could have introduced some kind of artifact, false positive or false negative.

The reviewer is correct in that we used Rh1-Gal4, and that Rh1 transcripts were found increased in *ninaE*^{G69D} photoreceptors. But we would like to highlight the fact that we validated the increases in *fabp* transcription through RT-PCR and western blots (Figure 1F, G). Because we have validated the results through independent methods, we are confident that increases in *fabp* transcripts in *ninaE*^{G69} photoreceptors is NOT due to artifacts in experimental design.

Conceptually, we do not think increases in *ninaE* expression would bias our transcription profiling experiments. This is because we pull down intact nuclei, where the ratio of individual mRNAs contained within those nuclei should remain unaffected by the strength of the Rh1-Gal4 activity. Any increases in Rh1-Gal4 would increase the amount of GFP:MSG expressed, but not the ratio of transcripts. As the reviewer may be aware, RNAseq analysis through the DESeq2 package examines the relative ratio of transcripts (through a normalization process). We now comment on this in the Materials and Methods section, "because the isolated nuclei remain intact until this stage, the ratio of the mRNAs within those nuclei should not be affected by any change in the levels of the Gal4 drivers" (page 21).

Among the identified genes, the authors report few ER stress related genes but we could have expected others such as Hsc-3/Bip or xbp-1. Could it suggest that this approach missed some of the genes upregulated?

My laboratory has conducted gene expression profiling studies in different tissues (including larval eye discs that is in press in eLife), and we find a certain degree of cell-type specificity in the UPR response. This is further reinforced by our published reporter studies, where UPR targets such as *4E-BP (Thor)* is induced in response to stress in one type of a tissue (e.g. fat body; Kang et al. (2017) *J Cell Biol* PMID 27979906), but not in another (e.g. photoreceptor cells). In our photoreceptor dataset, Hsc-3/BiP/Hsc70-3 was moderately induced, and we now mention this in the revised text page 7 "Another ER chaperone Hsc70-3 (also known as BiP), was induced at a more moderate level (Supplemental Table 1)."

-Rh1 levels accumulate in fabp mutant retina presumably due to a reduced light-dependent degradation of Rh1. This part is the weakest and it would be nice to provide some additional analysis to support that claim. First the authors should show that Rh1 transcripts are not affected in fabp mutants.

Thank you for the excellent point. We now include in the revised Figure 4C a q-RT-PCR data showing that Rh1 transcript levels do not change significantly in the *fabp* ^{EY02678} mutant background.

Regarding light-induced degradation of Rh1, the authors should test if fabp mutants affect Rh1 degradation by blue light exposure. This would strongly support their claim that fabp is implicated in light-induced Rh1 degradation.

We followed the reviewer suggestion and exposed flies to blue light for three hours before assessing Rh1 levels. We specifically examined three genotypes under these conditions: (1) *fabp wild type* control, (2) $fabp^{EY02678}$, and (3) $fabp^{EY02678}$; $Arr2^{3}$. The results have been added to the revised Figure S2, which supports the idea that *fabp* is required for Arr2-mediated Rh1 degradation in response to blue light exposure.

-Retinal degeneration in fabp is presumably due to the accumulation of Rh1 which when activated forms toxic complex with arrestin 2. The authors showed that degeneration is reduced in fabp ninaEG69D double mutant compared to single mutant which suggests that the reducing Rh1 level rescues fabp degeneration. Alternatively, fabp could reduce ER stress and protect from ninaEG69D-degeneration. To determine if indeed degeneration in fabp is due to Rh1 accumulation, the authors need to use the ninaEI17 null allele for the rescue.

We followed this excellent suggestion and added the results in the revised Figure 7C and D. The results show that reducing Rh1 through the introduction of $ninaE^{I17}$ dramatically delays the retinal degeneration of $fabp^{EY02678}$ flies ($p<0.0001$).

They should also perform a rescue for fabp degeneration in Vitamin A deficient food.

We understand that many *Drosophila* retinal degeneration studies examine vitamin A deficient conditions to assess the role of Rh1 proteins. However, we feel that this approach is inappropriate for our study, because vitamin A deficiency ALSO reduces *fabp* levels. Therefore, vitamin A deficiency experiments may cause more confusion in our case and make it difficult to interpret the results. We hope the reviewer agrees that the use of $ninaE^{I17}$ allele (in Figure 6E) addresses the point regarding the significance of Rh1 levels in retinal degeneration.

Finally, an interesting experiment would be to determine if arrestin2 mutant rescues also fabp degeneration.

Based on previous reports, *Arrestin2* mutants are not likely to rescue retinal degeneration in *fabp* mutants. Specifically, Dolph and colleagues reported that *Arrestin2* null mutants exhibit severe retinal degeneration just after 5 days of eclosion (Orem et al., 2006 PMID 16835270), because the photoreceptors have constitutive Rh1 signaling that can be toxic. Such retinal degeneration should be independent of that caused by excessive Rh1 in endosomes, because *Arrestin2* mutants block Rh1 endocytosis. Given the complexity, we feel that any retinal degeneration outcome of *Arrestin2; fabp* double mutants will be difficult to interpret.

-There is a possible issue related with the upregulation of Rh1 transcription in ninaEG69D and the use of Rh1-GFP in the quantification of neurodegeneration in Figure 6. The upregulation of Rh1-GFP may have introduced a bias in this analysis. I recommend to repeat this quantification by using retina semi thin sections to visualize and count the rhabodmeres.

In our revised Figure 7A-C and S3, we provide TEM images of *fabp* mutant eyes.
